# Nutrigonometry II: Experimental strategies to maximize nutritional information in multidimensional performance landscapes

**DOI:** 10.1002/ece3.9174

**Published:** 2022-08-04

**Authors:** Juliano Morimoto

**Affiliations:** ^1^ Institute of Mathematics King's College, University of Aberdeen Aberdeen UK; ^2^ School of Biological Sciences University of Aberdeen Aberdeen UK; ^3^ Programa de Pós‐graduação em Ecologia e Conservação Universidade Federal do Paraná Curitiba Brazil

**Keywords:** *Drosophila melanogaster*, fitness maps, lifespan‐reproduction trade‐off, nutritional geometry, trigonometry

## Abstract

Animals regulate their nutrient consumption to maximize the expression of fitness traits with competing nutritional needs (“nutritional trade‐offs”). Nutritional trade‐offs have been studied using a response surface modeling approach known as the Geometric Framework for nutrition (GF). Current experimental design in GF studies does not explore the entire area of the nutritional space resulting in performance landscapes that may be incomplete. This hampers our ability to understand the properties of the performance landscape (e.g., peak shape) from which meaningful biological insights can be obtained. Here, I tested alternative experimental designs to explore the full range of the performance landscape in GF studies. I compared the performance of the standard GF design strategy with three alternatives: hexagonal, square, and random points grid strategies with respect to their accuracy in reconstructing baseline performance landscapes from a landmark GF dataset. I showed that standard GF design did not reconstruct the properties of baseline performance landscape appropriately particularly for traits that respond strongly to the interaction between nutrients. Moreover, the peak estimates in the reconstructed performance landscape using standard GF design were accurate in terms of the nutrient ratio but incomplete in terms of peak shape. All other grid designs provided more accurate reconstructions of the baseline performance landscape while also providing accurate estimates of nutrient ratio and peak shape. Thus, alternative experimental designs can maximize information from performance landscapes in GF studies, enabling reliable biological insights into nutritional trade‐offs and physiological limits within and across species.

## Graphical abstract

In behavioral ecology, we have a powerful method, known as the Geometric Framework for Nutrition (GF), to study nutritional ecology. However, we have not yet, in the three decades since it was proposed, fully investigated whether its fundamental experimental design is likewise powerful. This study investigate the original and alternative sampling designs to reconstruct GF performance landscapes.

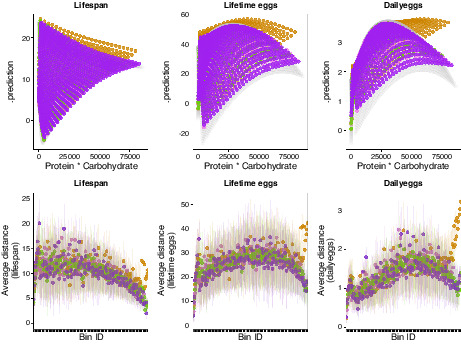

## 1 INTRODUCTION

Animals often balance their diet to maximize life‐history traits with diverging nutritional needs (Raubenheimer & Simpson, 2020; Simpson & Raubenheimer, 2012). This creates the potential for trade‐offs in the balance and allocation of nutrients needed for optimum fitness, aka “nutritional trade‐off” (Lee et al., 2008; Maklakov et al., 2008; Morimoto & Lihoreau, 2019). Nutritional trade‐offs have been described across taxa. For example, nutritional trade‐offs between lifespan and reproduction and between immunity and reproduction have been described in *Drosophila melanogaster* (Lee et al., 2008; Ponton et al., 2015), tephritid fruit flies and neriid flies (Adler et al., 2013; Fanson & Taylor, 2012; Fanson et al., 2012; Pascacio‐Villafán et al., 2022), crickets (Guo et al., 2022; Hawkes et al., 2022; Maklakov et al., 2008; Rapkin et al., 2018; Treidel et al., 2021), and mice (Solon‐Biet et al., 2014) (see also reviews by Ponton et al., 2011; Schwenke et al., 2016). Nutritional trade‐offs have also been described between reproductive traits in *D. melanogaster* (Morimoto & Wigby, 2016) and neriid flies (Sentinella et al., 2013), cockroaches (Bunning et al., 2015), crickets (Ng et al., 2018), and butterflies (Gage & Cook, 1994). Thus, nutritional trade‐offs appear to be ubiquitous.

A method known as the Geometric Framework of Nutrition (GF) has emerged as a powerful unifying framework capable of disentangling the multidimensional effects of nutrients (both ratios and concentrations) on life‐history traits and fitness (Raubenheimer & Simpson, 1993; Simpson & Raubenheimer, 1993), thereby enabling accurate estimates of nutritional trade‐offs. The GF framework has been used across taxa and became a cornerstone design for advancing our understanding of complex physiological and behavioral responses to nutrition, including human health (Raubenheimer et al., 2009; Simpson & Raubenheimer, 2012; Simpson et al., 2017). In essence, GF is an application of the response surface modeling (RSM) approach (Box & Wilson, 1951), where a *n‐*dimensional Euclidean space is used to investigate the response of the animal to the dietary intake of various ratios and concentrations of *n* nutrients. The resulting *n* + 1 surface (known as “performance landscape”) maps the level of the chosen trait across the different dietary ratios and concentrations. However, contrary to standard applications of RSM, GF is not only interested in optimization (i.e., finding the “peak” in the performance landscape). This is because the entire landscape contains valuable biological information about diet‐dependent expression of traits and thus, are meaningful to biologists and ecologists. For example, both peaks and valleys can be important indicators of the overall nutritional responses and comparisons between the positions of these properties within a performance landscape can be useful to determine the degree of changes in life‐histories with small dietary changes as well as quantifying obligate nutritional trade‐offs between traits (Alton et al., 2020; Kutz et al., 2019; Morimoto & Lihoreau, 2019; Rapkin et al., 2018). However, common design of experiments used in RSM such as full factorial or fractional designs and central composite designs (Myers et al., 2016) are not necessarily sufficient or efficient to reveal the characteristics of the entire performance landscapes (Ruohonen et al., 2001). Therefore, an optimum GF experimental design is a trade‐off between the number of diets and replicates per diet to maximize resolution of the performance landscape and the costs and feasibility risks associated with geometrically increasing sample sizes.

**FIGURE 1:**
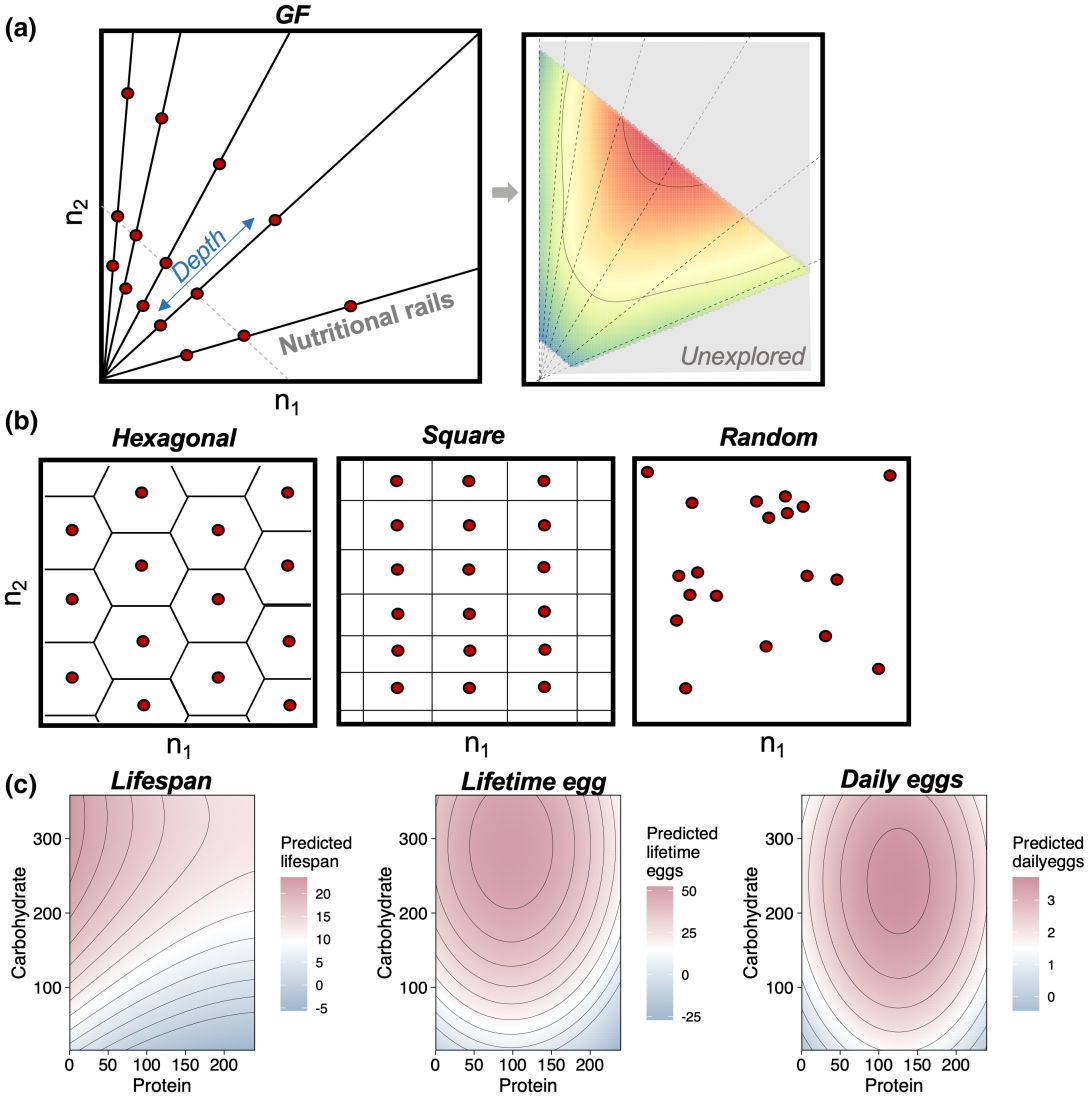
Exploration of the nutrient space using alternative sampling strategies. (a) The standard experimental design of a GF study (left) and a performance landscape generated form a fixed ratio dataset (reconstructed from Kutz et al., 2019) (right). Note the unexplored region in the nutritional space (shaded area). (b) The three alternative sampling strategies tested here: hexagonal, square, and random points grids. Red dots indicate anchor points (see Main Text). (c) The baseline performance landscapes for lifespan, lifetime egg production, and daily egg production. These landscapes were generated with the purpose of acting as the true performance landscape of the trait, which are unknown in GF experiments. These baselines landscapes are the standard upon which the reconstructed landscapes with alternative methods were compared against the GF in this study (see Methods section).

Traditionally, GF studies have been of two types: those which measure individual diet intake as in Fanson and Taylor (2012), Lee et al. (2008), Maklakov et al. (2008), and those that provide diets with fixed ratios and do not measure intake as in Alton et al. (2020) and Kutz et al. (2019). Both of these types share GF fundamental design of experiment which is as following: (i) the standard design of experiment in GF studies divides the nutritional space (i.e., Cartesian plane with nutrients as axes) into several “nutritional rails,” which are diets with fixed nutrient ratios (Figure 1a). (ii) each nutritional rail is subdivided into different diet concentrations. (iii) each combination of diet ratio and concentration (red dots in Figure 1a) are the “dietary treatments” which are given to replicate animals or group of animals, from where the measure of the traits are taken (Simpson & Raubenheimer, 2012); here, I will refer to the dietary treatment points as “anchor points” (Figure 1a). The difference between the two types of studies using GF is that on one type, experimenters measure individuals’ (or groups’) food intakes (“intake data”), whereas on the other type, individuals are given a fixed ratio of the diet without measurements of food intake (“fixed ratio data”). The anchor points (diets) are the points which contain data for the performance traits and therefore act as data‐driven points (or “anchors”) for the reconstruction of performance landscape, which is commonly done using thin‐plate spline interpolation (see e.g., Morimoto & Lihoreau, 2020; Ponton et al., 2015). Anchor points are directly used for interpolation in the fixed ratio data, but only work as guidelines for the experimental design for intake data, since the interpolation is done using the final nutrient intake of each individual in each diet. The performance landscape has depth determined by the variance in food intake (for intake data) or the range of diet concentrations (for fixed ratio data) (Figure 1a). Importantly though, both types of GF approaches are insufficient to generate anchor points that cover the entire area of the nutrient space, requiring interpolation while making performance landscapes incomplete. As a result, a large area of the nutrient space remains unexplored or in need of extrapolation for areas without anchor points (Figure 1a). While this may not necessarily affect our approximations of the region in which peaks and valleys are located, it certainly precludes us to extract meaningful biological information across the entire domain of the nutritional space of animals. For instance, by limiting the range of the nutrient space that is explored, GF makes an underlying assumption about the a priori knowledge of the physiological limits that a species has or evolved in terms of diet, although this information is seldomly known. More recent GF studies have used ecological and field work data to design GF diets that are ecologically relevant and guide experimental design, incorporating not only natural dietary information from natural populations (see e.g., Rothman et al., 2011; Vaudo et al., 2016; Wilder et al., 2013) but also genetics (Deans et al., 2016), environmental stability (Lawton et al., 2021), and land use (Le Gall et al., 2020). Despite this, GF studies are still inductive and do not explore the full range of the nutrient space. To date, there has been no systematic investigation as to how the standard GF experimental design can influence the resolution of the reconstructed performance landscapes, nor whether alternative experimental designs could provide more complete estimates of performance landscapes across the entire nutritional space.

Here, I investigated the performance of different sampling strategies when reconstructing performance landscapes, using a landmark dataset in the field of nutritional ecology (Lee et al., 2008). I used the pioneering Nutrigonometry framework to identify and compare the peaks in the reconstructed performance landscapes and how congruent these estimates are across sampling strategies (Morimoto et al., 2021). I tested four different sampling strategies: standard GF, hexagonal, squared, and random points sampling grids (Figure 1a). As a proof‐of‐concept, I developed the main arguments using fixed ratio datasets, as this type of GF approach is conceptually easier to explain and allows for the understanding of the foundations of my argument. I then expanded the applications of the argument for GF studies with intake datasets in the discussion section. Overall, this is the first investigation of the foundations of GF experimental design, which can have important long‐term implications to the quality of data collected in field of nutritional ecology. Expanding the coverage of performance landscapes will open up possibilities to extract biological information that is currently inaccessible, allowing for more complete studies on the nutritional trade‐offs that animals have evolved to circumvent physiological and nutritional constraints.

## 2 MATERIAL AND METHODS

### 2.1 Terminology and sampling designs

Throughout the text, I used the term “anchor point” to refer to a diet of given nutrient ratios and “resolution” as the total number of different diets (anchor points) of an experiment. Anchor points in the performance landscapes were generated in three resolutions: 30, 50, 250 anchor points (see Figure S1 for examples).

#### 2.1.1 Standard GF

Standard GF sampling grid was used with nutritional rails and diet concentrations as in the original dataset (Lee et al., 2008; Figure 1a).

#### 2.1.2 Hexagonal grid

The first alternative sampling strategy was the hexagonal grid (Figure 1b). Consider that knowledge (or “certainty”) $\eta_{i}$ about the estimate of the performance trait for trait *i* at the anchor point follows a (symmetric) Gaussian density function such that:

$$\eta_{i}=C*e^{\left(-\left[\frac{{\left(x-x_{0}\right)}^{2}}{2\sigma_{X}^{2}}\right]+\frac{{\left(y-y_{0}\right)}^{2}}{{2\sigma }_{Y}^{2}}\right)}$$

where *C* is amplitude of the distribution (e.g., determined by trait values), $x_{0}$ and $y_{0}$ are coordinates of the anchor points where the Gaussian is centered, and $\sigma_{X}^{2}$ and $\sigma_{Y}^{2}$ corresponds the uncertainty around the anchor point (Figure S2a). Note that for the purpose of this argument, I assume a correlation of zero between *x* and *y* and thus, a symmetric (circular) Gaussian. Then, the performance landscape can be seen as an analogous problem of circle packing in geometry, where the hexagonal grid is the densest circle packing in 2D Euclidean space (Chang & Wang, 2010) (see Figure S2b). In fact, the distance between any two anchor points *i* and *j* is equal to 2*r*, where *r* is the apothem of the hexagons containing the anchor points (see Figure S2c). I hypothesized that a hexagonal grid with anchor points at the center of hexagons could maximize performance landscape reconstruction in the nutritional space while minimizing the number of anchor points and replicates.

#### 2.1.3 Square grid

The second sampling strategy was the square grid (Figure 1b). The underlying rationale for the square grid is similar to that of the hexagonal grid above, where I divided the nutritional space in adjacent squares, with anchor points at the center of each square. The distance between any two anchor points *i* and *j* is equal to 2*r* if the squares lie in the same column or row and $2r\sqrt{2}$ if the anchor points lie in diagonal squares, where *r* is the apothem of the squares containing the anchor points (Figure S1c).

#### 2.1.4 Random points grid

Lastly, I also investigated the accuracy of a randomly probing the nutritional space (Figure 1b).

### 2.2 Dataset

I used a landmark dataset which contains *D. melanogaster* individual diet intakes and diet fixed ratios, and the consequences of diet on lifespan and reproduction (Lee et al., 2008). Two nutrients were investigated—protein and carbohydrate—such that performance landscapes have three dimensions. This dataset was previously used on my conceptualization of the Vector of Position approach and Nutrigonometry, having important benchmark status in the field (Morimoto & Lihoreau, 2019; Morimoto et al., 2021). Briefly, the Vector of Positions approach was developed to *n‐*dimensional performance landscapes from GF experiments as vector from which the strength of nutritional trade‐offs between traits can be estimated via the angle θ between vectors of two traits (Morimoto & Lihoreau, 2019). This approach uses a machine learning model to identify the peak region. More recently, I developed a conceptually simpler and computationally cheaper model to estimate peak regions and nutritional trade‐offs in GF studies using trigonometric relationships (“Nutrigonometry”), which enabled the comparison of different statistical methods to estimate nutritional trade‐offs and opened up new ways in which properties of performance landscapes can be estimated (Morimoto et al., 2021). The dataset used here was fundamental for the validation of these methods and is therefore used here.

### 2.3 Computation

#### 2.3.1 Generating the baseline performance landscape

The baseline performance landscape is the true performance landscape for the response of a trait throughout the nutritional space (Figure 1c). In experiments, this true performance landscape is unknown, and the GF framework aims to approximate a reconstructed performance landscape to the baseline landscape empirically. There are no available datasets in the literature which explores the entire nutritional space and thus, no true performance landscape have yet been estimated experimentally. Consequently, to obtain baseline performance landscapes, I created a high‐resolution grid (of 4 units distance between points) that covered the entire nutritional landscape including regions beyond the original boundaries of the nutritional space sampled by Lee et al., and predicted the value of the trait at each of the grid points using a machine learning approach based on the empirical values of the sampled regions obtained in Lee et al. (2008). This allowed me to create high‐resolution (predicted) baseline performance landscapes for lifespan, lifetime eggs, and daily eggs that can be compared with the reconstructed performance landscapes for the same traits using different sampling approaches (see below). The baseline landscapes are shown in Figure 1c.

#### 2.3.2 Approach

I simulated a real‐world experiment as following: (i) I sampled the anchor points in the nutritional space according to the four grid sampling approaches and three resolutions tested here (see above). (ii) I used a polynomial regression with the linear and quadratic effects of protein and carbohydrate (and their interactions) fitted to the baseline performance landscape to assign a value of the trait to each of the anchor points. This is equivalent to running an experiment with the anchor points and obtaining the estimates for the performance trait at each anchor point, based on the true performance landscape (which is unknown in real world problems). (iii) I reconstructed the performance landscape for each sampling grid and resolution using the thin‐plate spline method. (iv) I applied the Nutrigonometry model to estimate the peak region for each sampling grid and resolution on the reconstructed landscape, which included the calculation of the protein‐to‐carbohydrate (P:C) ratios of the estimated Nutrigonometry peak. (vi) I overlaid the identified peak region with the true performance landscape as well as all the estimates of peak region for across all resolutions and grid sampling strategies. I calculated the area of the estimated peak by approximating the area to an ellipse that had coordinates determined by the peak estimates using Nutrigonometry.

#### 2.3.3 Reconstruction accuracy

I estimated accuracy of the reconstruction vs baseline landscape by generating a 2D profile of the 3D landscape by using the predicted value of the landscape as *y*‐axis and the multiplication of protein and carbohydrate content as *x*‐axis, for all points in the landscape. This approach allowed for dimensionality reduction while providing topological information of the structure of the data. I then binned the *x*‐axis (*n* = 100) and calculated the average and standard deviation of the Euclidean distance between the points from the baseline performance landscape and the reconstructed performance landscape. For this analysis, I used the reconstructed landscapes with highest resolution (i.e., 250) because of the higher density of anchor points (and hence, expected accuracy) used to generate these performance landscapes.

### 2.4 Software and packages

All simulations were performed in R version 3.6.2 (R Core Team, 2019). The “tidymodels version 0.1.0,” “stringr version 1.4.0,” “tidyr version 1.1.0,” “purrr version 0.3.4,” and “dplyr version 0.8.5” packages of the tidyverse were used for data wrangling, as well as to generate the baseline performance landscape and manipulate data for visualization (Wickham et al., 2019). Performance landscapes were reconstructed using the “Tps” function of the “fields version 10.3” package with lambda argument set to 0.05 in all models (Nychka et al., 2017). I also used the “raster version 3.1‐5,” “rgeos version 0.5‐3” and “sp version 1.4‐2” packages for data manipulations for visualization and sampling of the nutritional space, the latter being used for the functionalities in the “spsample” function (Bivand et al., 2017; Hijmans et al., 2022; Pebesma & Bivand, 2005). All plots were done using the “ggplot2 version 3.3.1” package (Wickham, 2016). The “ggnewscale version 0.4.5” package was used to prettify the data visualization (Campitelli, 2020).

## 3 RESULTS

### 3.1 Standard GF sampling strategy finds the correct ratio of nutrients but often with inaccurate peak shape estimates

**FIGURE 2:**
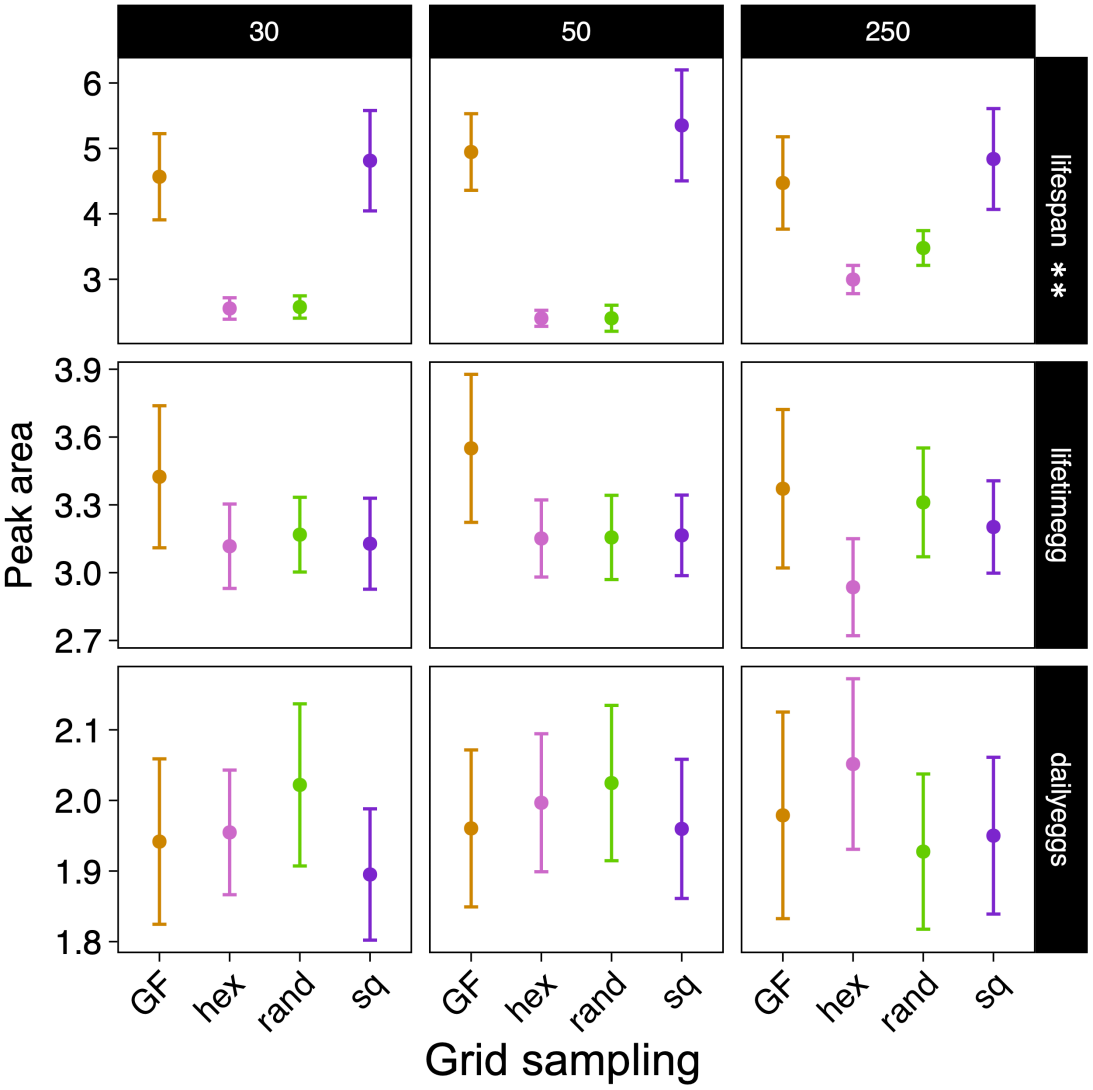
P:C ratios of the estimated peak in the reconstructed performance landscape across the grid sampling strategies. ^**^Note that the *y*‐axis of the lifespan plots was log‐transformed to aid data visualization (see also Figure 3a). Such differences in scale for lifespan emerged from the fact that the peak lies near the boundary of the nutritional landscape, in a region of P:C ~ 0:1. Hex, hexagonal sampling; rand, random points sampling; sq, square sampling.

**FIGURE 3:**
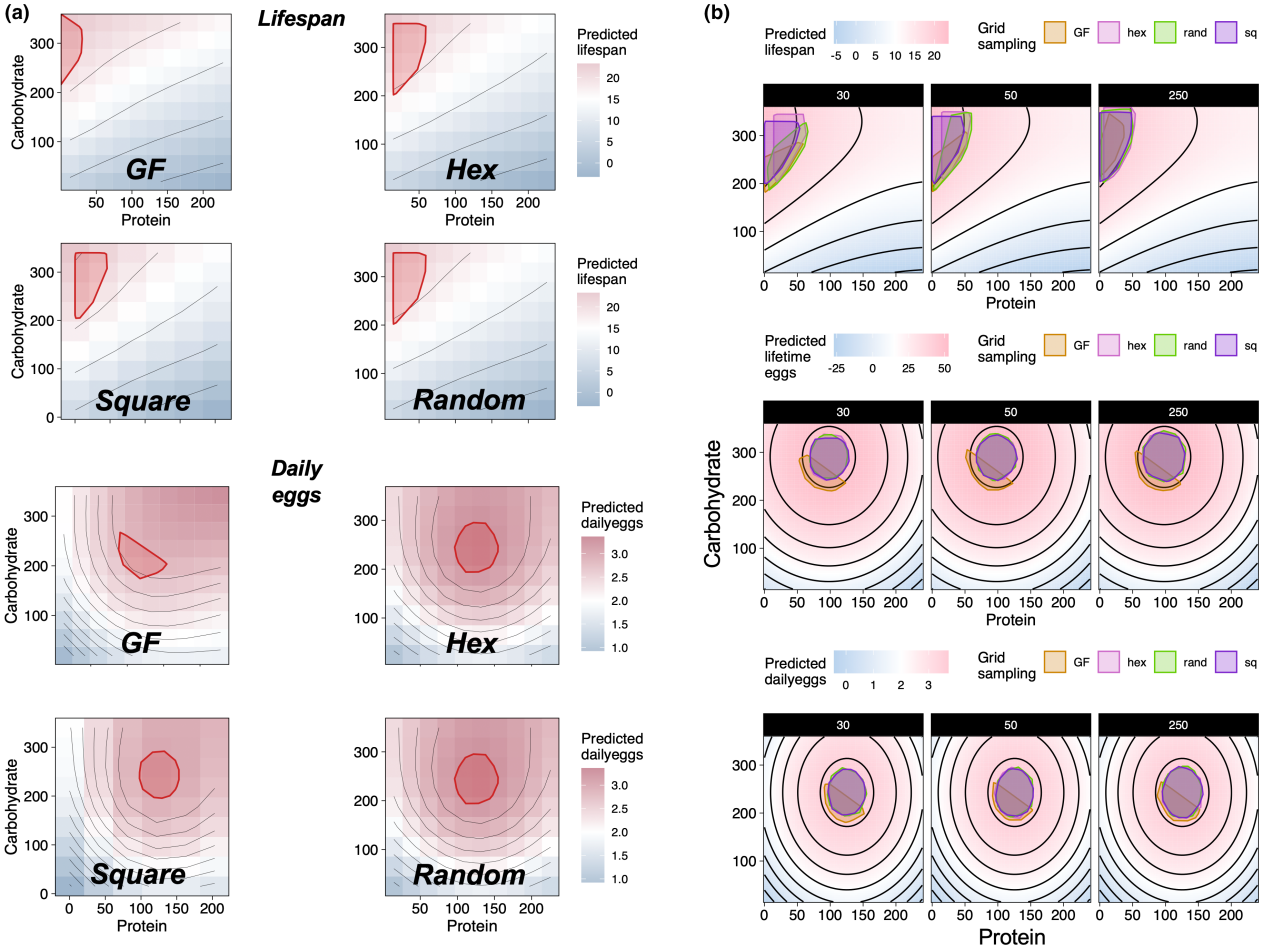
Predicted peak region and shape across sampling strategies. (a) Predicted peak in the performance landscape of lifespan (top) and daily eggs (bottom) (see also Figure S1d for lifetime egg peak predictions). Performance landscapes reconstructed from resolution equal to 50. (b) Overlaid peak predictions mapped onto the baseline performance landscapes of lifespan, lifetime eggs, and daily eggs across the sampling strategies. Note that GF sampling (orange) generates incomplete peak shape predictions for traits that respond to the interaction of nutrients. Hex = hexagonal sampling; rand = random points sampling; sq = square sampling.

**FIGURE 4:**
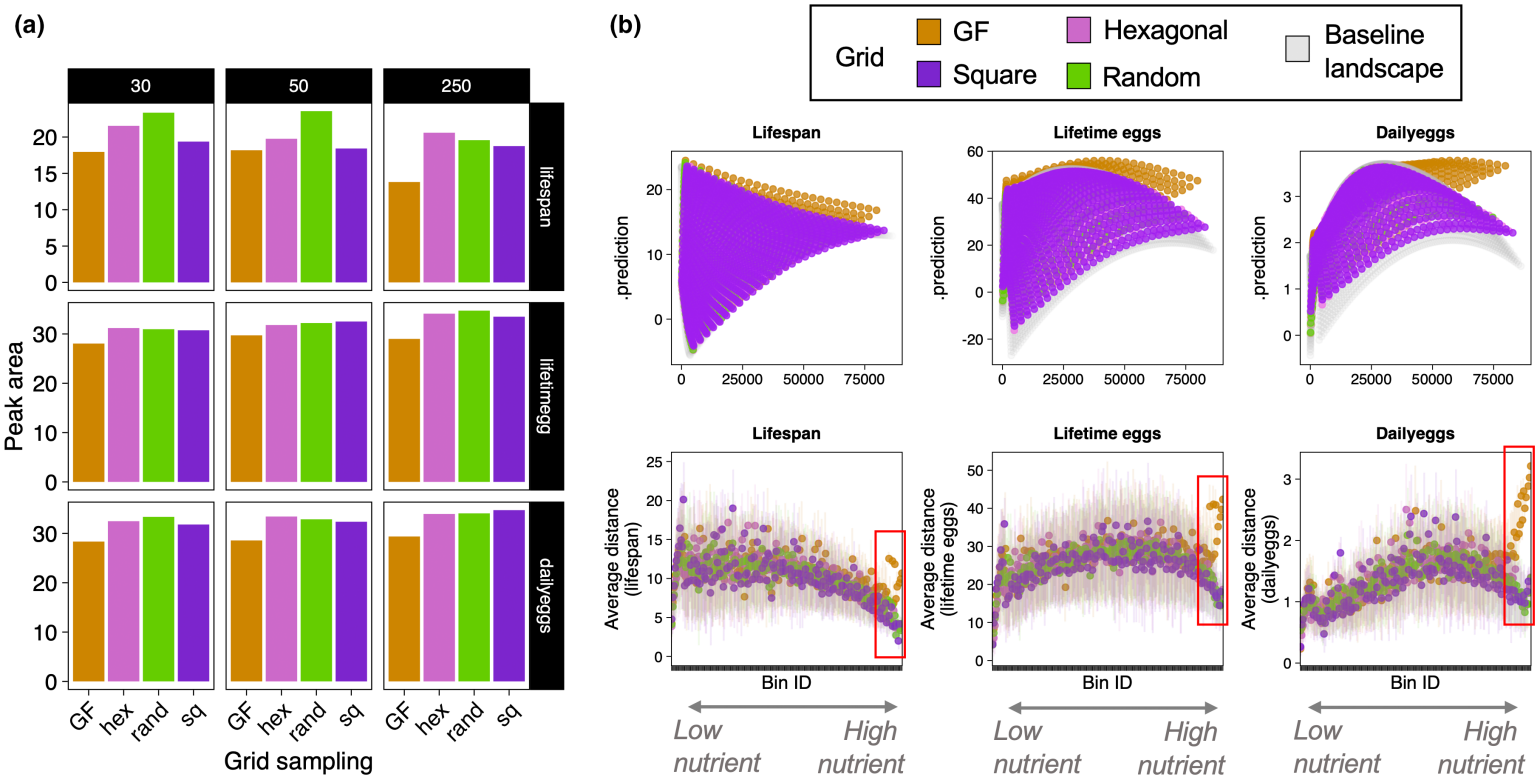
Peak area and performance landscape topology. (a) Predicted peak area (i.e., area of the shaded polygon from the predicted region for lifespan and reproductive rate data), with structure containing individual intakes. (b) Distance between the underlying landscape (faded black) and the reconstructed landscapes from different grid sampling strategies. Note that the average distance increases for GF sampling strategy (orange) in regions of high nutrient intake, and this distance is particularly accentuated when the underlying landscape has a peak in the middle of the performance landscape indicating interactions between nutrients (red boxes).

The data showed that all sampling strategies provide reasonably accurate estimates of the ratio in which the peak in the performance landscape is found (Figure 2). Note that estimates of peak P:C ratio were more variable for lifespan (log_10_‐transformed in Figure 2 to aid visualization) because the peak lies near the boundary of the performance landscape (i.e., P:C ~ 0:1). Nonetheless the visualization of the predicted peak region shows that all methods find peak regions in the correct area of the performance landscape (Figure 3a). Despite this, striking difference between standard GF and other sampling strategies were found in the shape of the predicted peak. For lifespan, where the peak in the performance landscape lies near the boundary of the nutritional space, the predictions of all sampling strategies were similar in shape (Figure 3a,b). Conversely, the shapes of the GF peaks for lifetime egg production and daily eggs, which are in the middle of the performance landscape indicating that the trait responds to the interaction between protein and carbohydrate, differed substantially from that of other sampling strategies: standard GF peaks are wider and semi‐circular while all other sampling strategies find a defined circular peak covering the appropriate region of the baseline performance landscape (Figures 3a,b and S1d). The overlaid visualization of the peak estimates and the underlying baseline performance landscape clearly showed that estimates of peak region from GF sampling were incapable of reflecting the true peak region of the baseline performance landscape relative to the other methods (Figure 3b). As a result, these differences are also reflected in the peak area estimates where the peak area using standard GF was smaller relative to other sampling strategies (Figure 4a). In other words, standard GF can only provide a partial estimate of peak area, especially for traits that are affected by the interaction between nutrients.

### 3.2 Reconstructed performance landscapes from standard GF sampling are more inaccurate in regions that capture responses to nutrient interactions

The topological profile of the reconstructed landscapes showed that standard GF sampling generates reconstructed performance landscapes more dissimilar (measured as the Euclidean distance) to the true baseline performance landscape in regions that capture the interaction of nutrients on the performance trait (e.g., high protein and high carbohydrate values) (Figure 4b). Importantly, the inaccuracy is less accentuated for traits that have peak near the boundary of the nutritional space (i.e., lifespan), but progressively more pronounced for traits with peak in the middle of the nutrient space, which indicates strong responses to the interaction of nutrients (Figure 4b). For instance, in regions of high protein and carbohydrate, the average Euclidean distance between the reconstructed and true baseline performance landscapes increases rapidly for standard GF sampling relative to other sampling strategies, particularly for the landscapes of lifetime eggs (with peak at P:C ~ 1:3) and daily eggs (P:C ~ 1:2) (see highlighted red region in Figure 4b).

## 4 DISCUSSION

I report an investigation of alternative sampling strategies of the nutritional space for GF studies. This is necessary so that GF landscapes can be made more robust, from which properties can be estimated and biological insights, gained. This goes above and beyond current efforts that integrated ecological information into the design of traditional GF studies as those suffer from similar limitations that underpinned this work (i.e., regions of the nutrient space with a lack of sampling) (Rothman et al., 2011; Vaudo et al., 2016; Wilder et al., 2013). I tested three alternative grid sampling strategies: hexagonal, square, and random points grids. Using a landmark dataset coupled with the pioneering Nutrigonometry method, I showed that all sampling strategies are able to provide reasonable estimates of the nutrient ratios where the peak in nutritional landscape is found. However, GF sampling provides incomplete estimates of peak region. This can have knock‐on consequences for biological inferences when, for example, peak area is relevant to understand the nutritional conditions which maximize the expression of a trait. Importantly, GF sampling also provides inaccurate estimates of the performance landscape shape for performance traits that respond to the interaction between nutrients, highlighting additional limitations of the standard GF experimental design for biological insight using the properties of the performance landscapes. Overall, this study shows that to build performance landscapes with reliable shapes for biological inferences, alternative strategies of experimental design are needed in GF studies.

Why does the GF sampling find the correct information of nutrient ratios but not on the shape of the peaks in the landscape? Figure 1a (right panel) shows that the GF sampling strategy explores only a subset of the nutritional space. For fixed ratio datasets, this is usually a triangular region, whereas for intake datasets, the shape can vary, but never covers the entire nutrient space. As a result, the interpolation for the construction of the performance landscape becomes an *extrapolation* beyond the regions upon which the anchor points exist, which can be mathematically and computationally difficult to achieve even with more complete datasets (Campagna & Perracchione, 2021). As a result, the standard thin‐plate‐spline interpolation and subsequent algorithms to estimate peak position truncate the peak estimates on the boundary of the performance landscape that can be estimated based on the anchor points. In doing so, the shape and area of the peak is also truncated, losing important biological information (Figure 3). The alternative methods tested here circumvent this limitation by sampling a wider range of the nutrient space, with anchor points that support a more accurate estimate of the peak shape and area.

Why does the GF sampling lead to more inaccurate landscapes in regions of nutrient interactions? The first reason lies on the previous point: GF only covers a subset of the nutritional space. Often, the diagonal region of the nutrient space has less “covered area” relative to empty nutrient space (see e.g., Figure 1a). Consequently, a larger area of the performance landscape is missing and needs to be extrapolated, which can result in higher error. The second reason is likely related to the curvature of the performance landscape. I showed that the inaccuracies increase in performance landscapes for traits with peaks in the middle of the nutrient space, which indicates that the trait responds to the interaction between nutrients rather than an additive effect. For instance, the inaccuracies were almost absent for the landscape of lifespan, but progressively more accentuated for the landscapes of lifetime eggs and daily eggs, respectively (Figure 4b). The absence of anchor points (i.e., diets) covering the full diagonal region likely precludes an adequate estimate of the curvature of the performance landscape in regions of nutrient interactions. The alternative methods tested here circumvent both of these limitations of GF sampling by covering a wider region of the nutrient space, including in the diagonal region. Note, however, that although the alternative methods perform better than standard GF sampling, they still introduce inaccuracies in the performance landscapes in the regions of nutrient interactions, providing an important area for future theoretical, computational, and empirical work to understand the underlying reasons.

**FIGURE 5:**
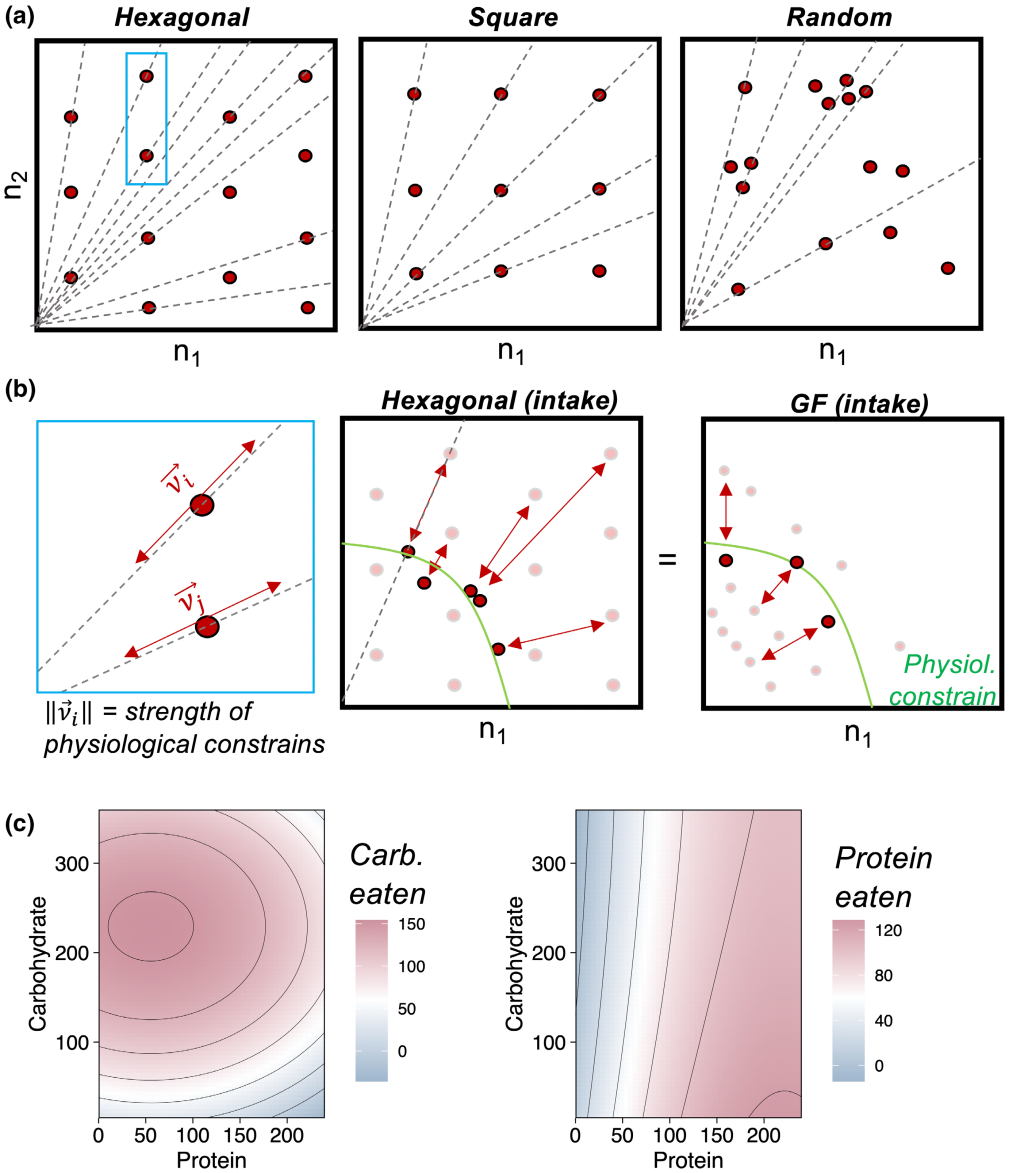
Alternative sampling strategies used for intake datasets. (a) Each anchor point could be seen as a point in a nutritional rail (as defined in standard GF design). This is true for all alternative sampling strategies tested in this study. (b) (left) Zoom of a specific region of the nutritional space from the hexagonal grid strategy (in *a*). When measuring intake, the anchor points move along nutritional rails represented by a vector $\vec{\nu_{i}}$ for the *i*th anchor point. The magnitude of the vector, $\left\|\vec{\nu_{i}}\right\|$, provides a measure of the strength of the physiological constraint experienced by the animals across diets as this metric shows the distance traveled by the anchor point along the nutritional rail (center and right panels). Green line represents a hypothetical demand imposed by physiological constraint. (c) Performance landscapes of protein and carbohydrate intake (from Lee et al., 2008) to illustrate how intake can be used as the third dimension in performance landscapes. This can assist the inferences of rules of compromise which determine the amount of food and the quantity of each nutrient that individuals are capable of over‐ or under‐consume in order to minimize distance between current food intake and self‐balanced food intake (Raubenheimer & Simpson, 1993; Simpson & Raubenheimer, 1993). Rules of compromise are not dealt with in this study as it lies beyond the study’s main scope, and is part of a next manuscript of this series.

In this study, I used fixed ratio datasets as a proof‐of‐concept, which is the structure that has been used recently in studies of GF focused on development (Alton et al., 2020; Kutz et al., 2019; Ma et al., 2020; Silva‐Soares et al., 2017), but GF sampling primarily covers datasets with individual nutrient intakes (e.g., Hawkes et al., 2022; Lee et al., 2008; Maklakov et al., 2008). Individuals' nutrient intakes are constrained by animal physiology and are difficult or impossible to overcome (e.g., individuals often die in overly unbalanced diet). Consequently, animals will unlikely eat sufficient quantities of food to explore the entire nutrient space, particularly in diets that are highly unbalanced relative to physiological constraints. As a result, the anchor points will be shifted in the direction of the physiological constraint, which can be represented by a vector ${\vec{\nu }}_{i}$ (Figure 5a,b). Note that each anchor point can be represented as a point in a nutritional rail, which determines the direction of the vector ${\vec{\nu }}_{i}$ (Figure 5b). In this case, the anchor points for any performance landscape of the alternative sampling strategies tested here, if plotted using intakes, will yield a similar performance landscape to that generated by GF sampling because individuals will shift their intakes to match the physiological constraints (Figure 5b). In other words, the performance landscapes from all methods will tend to converge. This is important because nutrient intake data can reveal physiological constraints as well as compensatory feeding strategies underpinning rules of compromise, where individuals modulate the intake of more (or less) concentrated diets to achieve similar P:C ratios and total nutrient intake (Raubenheimer & Simpson, 1993). This information is unavailable in fixed ratio data where intake is not measured. Several questions could be raised, for instance: (i) how can the limitations of alternative methods in terms of representing nutrient intakes to derive rules of compromise be resolved? Or (ii) why then, use alternative methods, if they either fail to provide intake datasets *or* converge toward the standard GF sampling strategy? To answer the first question, it is important to notice that it is not mandatory to use nutrient intakes to define the anchor points when generating landscapes (e.g., Alton et al., 2020; Kutz et al., 2019). It is true that in general, GF studies have used individual nutrient intake as an *input* variable upon which the performance trait was mapped and the landscape built (see Simpson & Raubenheimer, 2012 for a comprehensive review). However, intake estimates can and have been used as the *output* (performance) variable in GF studies, opening up the possibility of using fixed ratios for the design of experiments and nutrient intake as performance traits (rather than input variables). For example, a GF study showed that yeast‐rich diets induce higher water intakes in *D. melanogaster* (Fanson et al., 2012). That said, it is possible to use the alternative methods presented here as fixed ratios upon which nutrient intake and performance traits can be mapped (Figure 5c). I conjecture that this approach will enable us to extract the same rules of compromise and insights into physiological constraints as the original GF approach. The formalization of this conjecture requires an extensive argument that lies beyond the scope of this paper as it involves introducing new concepts, for example, intake targets (Simpson & Raubenheimer, 2012), but is part of a follow‐on manuscript being conceived. Importantly, the conjecture must be valid under the assumption that performance landscapes of alternative methods and GF sampling are to converge (as in Figure 5b). This leads to the answer of the second question: why then use alternative methods? Alternative methods allow for more complete exploration and accuracy in the representation of performance landscapes, as shown here. This opens up the possibility to use properties of the performance landscapes as new proxies for biological insights. For instance, peak area and shape could provide insights into the nutritional resilience of the animal in maximizing a trait under varying nutritional conditions (e.g., the wider the peak, the more nutritionally resilient the animal). The use of the properties of the performance landscape cannot be achieved unless performance landscapes explore the entire nutritional space. Thus, alternative sampling methods expand the scope of GF methods and can unlock new measurements that can provide unique insights into compensatory feeding strategies with biological significance and more broadly, the evolution of nutritional trade‐offs (Fanson et al., 2012; Raubenheimer & Simpson, 1993).

## 5 CONCLUSION

Despite the growing integration of ecological information into experimental design, current GF studies use a design aimed at sampling the nutrient space to construct performance landscapes that had not been scrutinized (Deans et al., 2016; Lawton et al., 2021; Le Gall et al., 2020; Rothman et al., 2011; Vaudo et al., 2016; Wilder et al., 2013). I tested alternative sampling strategies and show that their performances in reconstructing landscape's properties are superior. From these alternative strategies, the hexagonal design seems the most obvious choice for empirical test as it allows for anchor points to be distributed such that more area is covered in the nutrient space per anchor point. Future studies will illuminate how other standard metrics calculated in GF studies (i.e., rules of compromise) can be estimated and calculated from fixed ratio data with hexagonal (or other sampling strategy) design. This includes for instance regions in which the combination of nutrients are potentially lethal, generating holes in the performance landscapes (Blonder, 2016; Conceição & Morimoto, 2022). Overall, the findings shown represent an advance to current GF experimental design methodology. This has important consequences to the field because GF enables a multidimensional approach in nutrition where performance landscapes can provide important biological insights into the evolution of animal nutrition and life‐histories.

## AUTHOR CONTRIBUTIONS

**Juliano Morimoto:** Conceptualization (equal); data curation (equal); formal analysis (equal); investigation (equal); methodology (equal); project administration (equal); resources (equal); validation (equal); visualization (equal); writing – original draft (equal); writing – review and editing (equal).

## CONFLICT OF INTEREST

The author has no conflict of interests to declare.

## Supporting information


Text S1
Figure S1
Figure S2
R Script

## Data Availability

Lee et al. 2008 dataset accompanies the R code and is also available in Dryad: https://doi.org/10.5061/dryad.tp7519s. R script with functions for the implementation of the analysis will be made available in available as supplementary material.
