# Supplementary material for: Nutrigonometry II: Experimental strategies to maximize nutritional information in multidimensional performance landscapes
Source: Ecol Evol. 2022 Aug 4;12(8):e9174. doi: 10.1002/ece3.9174 (PMC9353123; doi:10.1002/ece3.9174)
Supplement: Supplementary file 1 — Text S1 Figure S1 Figure S2 R Script [file ECE3-12-e9174-s001.zip › ece39174-sup-0001-FiguresS1,S2.docx]

**Supplementary Material: “Nutrigonometry II: experimental strategies to maximise nutritional information in multidimensional performance landscapes”**

Author: Juliano Morimoto^1,2,3*^

Author’s affiliations:

^1^ Institute of Mathematics, University of Aberdeen, King's College, Aberdeen AB24 3FX

^2^ School of Biological Sciences, University of Aberdeen, Zoology Building, Tillydrone Ave, Aberdeen AB24 2TZ

^3^Programa de Pós-graduação em Ecologia e Conservação, Universidade Federal do Paraná, Curitiba, 82590-300, Brazil

* Correspondence:

Dr Juliano Morimoto

[juliano.morimoto@abdn.ac.uk](mailto:juliano.morimoto@abdn.ac.uk)

**Running-title:** GF sampling strategies

**Keywords**: Nutritional Geometry, trigonometry, lifespan-reproduction trade-off, fitness maps, *Drosophila melanogaster*

**Figure S1.** (a-c) Examples of the sampling strategies for resolutions of 30 (a) 50 (b) and 250 (c). (d) Prediction of the peak regions for lifetime egg across sampling strategies [data from Lee et al. (2008)].

**Figure S2.** (a-b) Analogy to the circle packing problem and 2D Gaussian function as a way to determine certainty of performance trait information. (c) The distances between the anchor points in a hexagonal and square grid.

**R Script.** R script with functions for the implementation of the Nutrigonometry framework (separate file).
